# Modelling brain dopamine-serotonin vesicular transport disease in *Caenorhabditis elegans*

**DOI:** 10.1242/dmm.035709

**Published:** 2018-11-09

**Authors:** Alexander T. Young, Kien N. Ly, Callum Wilson, Klaus Lehnert, Russell G. Snell, Suzanne J. Reid, Jessie C. Jacobsen

**Affiliations:** 1 The School of Biological Sciences and Centre for Brain Research, The University of Auckland, Auckland 1010, New Zealand; 2 Adult and Paediatric National Metabolic Service, Auckland City Hospital, Auckland 1023, New Zealand

**Keywords:** VMAT, Cat-1, Dopamine-serotonin transport disease, *Caenorhabditis elegans*, Animal model

## Abstract

Brain dopamine-serotonin vesicular transport disease is a rare disease caused by autosomal recessive mutations in the *SLC18A2* gene, which encodes the VMAT2 protein. VMAT2 is a membrane protein responsible for vesicular transport of monoamines, and its disruption negatively affects neurotransmission. This results in a severe neurodevelopmental disorder affecting motor skills and development, and causes muscular hypotonia. The condition was initially described in a consanguineous Saudi Arabian family with affected siblings homozygous for a P387L mutation. We subsequently found a second mutation in a New Zealand family (homozygous P237H), which was later also identified in an Iraqi family. Pramipexole has been shown to have some therapeutic benefit. Transgenic *Caenorhabditis elegans* were developed to model the P237H and P387L mutations. Investigations into dopamine- and serotonin-related *C. elegans* phenotypes, including pharyngeal pumping and grazing, showed that both mutations cause significant impairment of these processes when compared with a non-transgenic N2 strain and a transgenic containing the wild-type human *SLC18A2* gene. Preliminary experiments investigating the therapeutic effects of serotonin and pramipexole demonstrated that serotonin could successfully restore the pharyngeal pumping phenotype. These analyses provide further support for the role of these mutations in this disease.

## Abstract (precis)

**Summary:** The first *Caenorhabditis elegans* model to study brain dopamine-serotonin vesicular transport disease, demonstrating impairment of pharyngeal pumping and grazing phenotypes.

## INTRODUCTION

Brain dopamine-serotonin vesicular transport disease is a rare neurological disease caused by mutations in the vesicular monoamine transporter 2 (VMAT2; encoded by *SLC18A2*), an H^+^ ATPase antiporter required for transport of neurotransmitters into presynaptic vesicles. This disease was first reported in 2013, in eight members of a consanguineous Saudi Arabian family who carry an autosomal recessive mutation in *SLC18A2* (OMIM: 193001, Refseq NM_003054.4; P387L, c.1160C>T) (Rilstone et al., 2013). Two further cases harbouring homozygous recessive mutations have since been reported in our previously described New Zealand family (P237H, c. 710C>A) (Jacobsen et al., 2016) and an Iraqi family with the same mutation (Rath et al., 2017). The two mutations identified for this disorder are in evolutionarily conserved positions and lie within transmembrane domains 5 and 10 of VMAT2, respectively.

Affected individuals display truncal hypotonia and have impaired motor skills. Other symptoms include lack of head control, uncontrolled eye movements, abnormal posturing, tremor, dysdiadochokinesia, dysarthria, dystonia and parkinsonism, as well as oropharyngeal and nasal secretions, extreme sweating, fatigue, poor distal circulation, sleep disruptions and hypernasal speech. However, there is some diversity in the phenotypic spectrum of the disease across the three published cases (Jacobsen et al., 2016; Rath et al., 2017; Rilstone et al., 2013). Remarkably, all three reports describe various degrees of improvement after treatment with the dopamine agonist pramipexole [a dopamine D2/D3 receptor preferring agonist (Kvermo et al., 2006)], including improved parkinsonism, gaining control of eye movement, improved fine motor skills, improved learning ability and gaining the ability to walk (Rath et al., 2017; Rilstone et al., 2013). Interestingly, cases treated at an earlier age responded better to pramipexole; for example, the therapeutic improvement in the New Zealand case (at age 14) was not as remarkable as the Iraqi case (at age 7) despite harbouring the same mutation (Jacobsen et al., 2016). Interestingly, patients treated with the dopamine precursor L-DOPA demonstrated worsening of symptoms (Jacobsen et al., 2016; Rilstone et al., 2013).

To investigate the effects of the original P387L mutation, Rilstone et al. expressed wild-type (WT) and mutant VMAT2 in COS-7 cells (Rilstone et al., 2013). The mutant VMAT2 demonstrated reduced serotonin signalling, but glycosylation of the protein was not affected, indicative of normal protein processing. Furthermore, serotonin signalling in mutant VMAT2 was slightly higher than WT VMAT2 inhibited with reserpine (a VMAT2 blocker), suggesting that the mutation does not result in complete loss of function (Rilstone et al., 2013).

To expand on the functional work by Rilstone et al. and support the role of the P237H mutation in disease, we developed *Caenorhabditis elegans* models of brain dopamine-serotonin vesicular transport disease. *Caenorhabditis elegans* is a nematode that is commonly used as a simple multicellular model of human diseases. *Caenorhabditis elegans* exist predominantly as female hermaphrodites with very few males, and can develop in 3 days from eggs to adults. Self-fertilising adults can produce about 300 progeny, enabling access to very large populations and making them a simple, cost-effective option for studying the biology of neuronal signalling. *Caenorhabditis elegans* have a VMAT homologue, *cat-1*, which has 49% amino acid sequence similarity to human VMAT2, with higher regions of conservation in the transmembrane domains where the mutations reside. *cat-1* is located on the X chromosome, and is expressed in all dopamine- and serotonin-containing cells in *C. elegans*. Like human VMAT2, *C. elegans* VMAT is responsible for the transport of dopamine and serotonin into synaptic vesicles (Duerr et al., 1999). *Caenorhabditis elegans* lacking VMAT are viable and grow well, but are deficient for dopamine- and serotonin-associated behaviours, suggesting that neurotransmitter function is dependent on synaptic vesicle loading (Duerr et al., 1999). We subsequently generated three transgenic lines – *cat-1(−)-huVMAT2(+)*, *cat-1(−)-huVMAT2(P387L)* and *cat-1(−)-huVMAT2(P237H)* – to assess the impact of these mutations on VMAT2 function.

## RESULTS

### Transgenic strain development

**Fig. 1.**
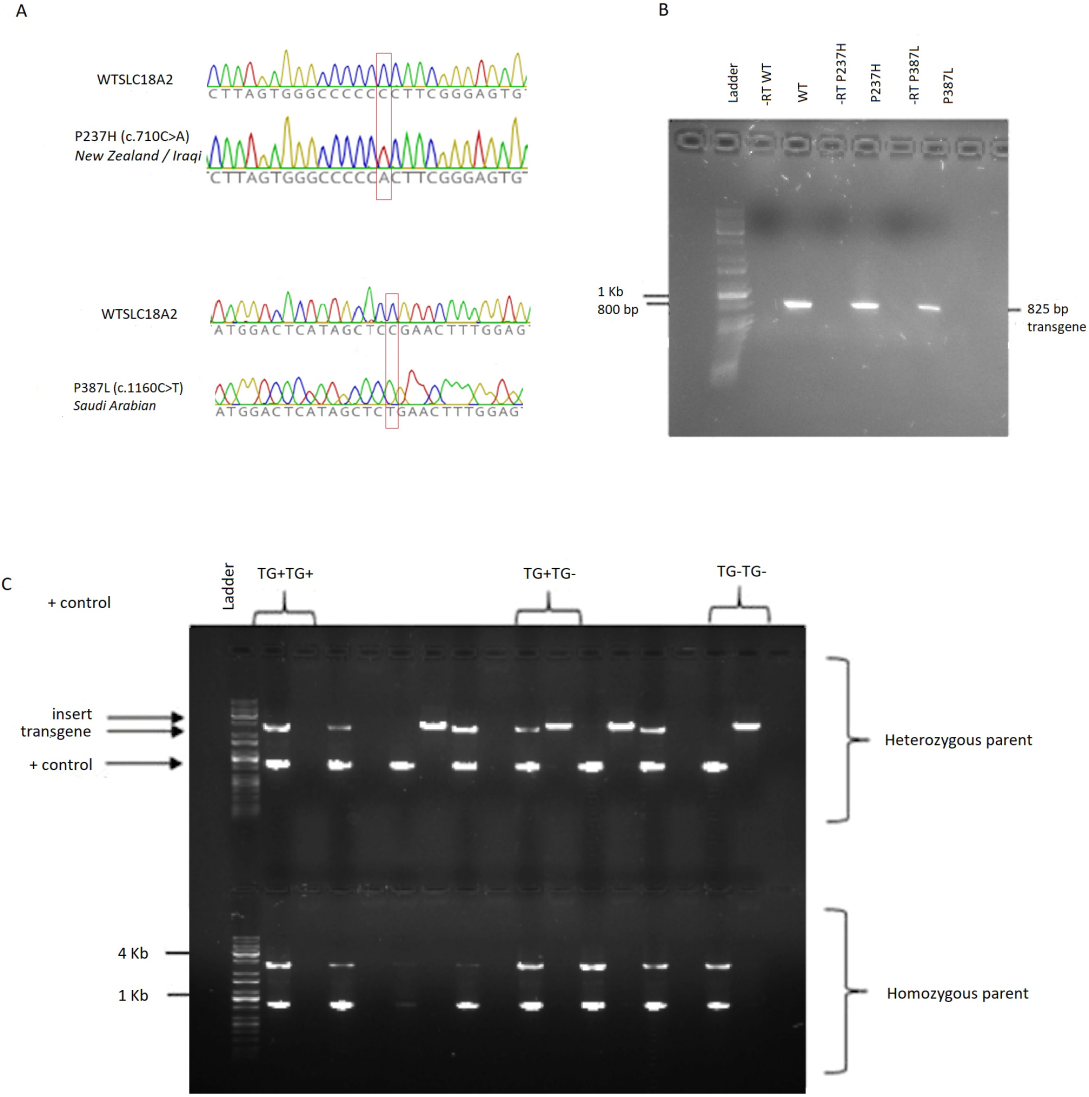
**Confirmation of transgenics.** (A) Sanger traces confirming that the mutations are present in the transgenes integrated into the *C. elegans* strains. Mutation sites are indicated by red boxes. (B) Confirmation of gene expression by RT-PCR (primers seqhVMATf1 and seqhVMATr1). RT negative controls contained no reverse transcriptase. P237H (NZ), P387L (SA), WT. (C) Prior to Sanger sequencing, *C. elegans* were genotyped by PCR. Each *C. elegans* required 2 wells for assessment: the first well contains a positive control (SSU18A+SSU26R ∼950 bp) and a test for the transgene (primers unc5402 and seqSNB01, 2657 bp). Primers NM3884 and NM3880, which flank the integration site, were used to determine insertion of the construct. The second well for each sample only show a product (2945 bp) if one chromosome does not have the construct inserted, and no band if the *C. elegans* is homozygous. The top series of bands are all from *C. elegans* of a single parent, deemed heterozygous, as all possible genotype combinations are represented. The lower bands are from a homozygous parent, as all *C. elegans* show presence of the transgene, and a copy on each chromosome. Primer sequences are available in Table S3. TG, transgene.

Three transgenic *C. elegans* lines were successfully generated using the MosSCI method: *cat-1(−)-huVMAT2(+)*, which is homozygous for the WT human *SLC18A2* gene; *cat-1(−)-huVMAT2(P237H)*, carrying the *P237H* (c.*710C>A*) human mutation; and *cat-1(−)-huVMAT2(P387L)*, carrying the *P387L* (*c.1160C>T*) human mutation. The genotype was confirmed using polymerase chain reaction (PCR; Fig. 1C), and the transgenic status of the final strains was confirmed by Sanger sequencing (Fig. 1A). Successful transcription of the transgene was verified using reverse transcription PCR (RT-PCR; Fig. 1B). The crossing method only selected for *cat-1* knockouts, which was confirmed by Sanger sequencing (Fig. S2).

The transgenic *C. elegans* lines were subsequently assessed for dopamine- and serotonin-dependent behaviours.

### Phenotyping

#### Grazing

Grazing behaviour is a well-established phenotype in *C. elegans*, and is known to be dependent on both dopamine and serotonin (Chase and Koelle, 2007). It was therefore used to assess the effect of both mutations on dopamine and serotonin signalling.

**Fig. 2.**
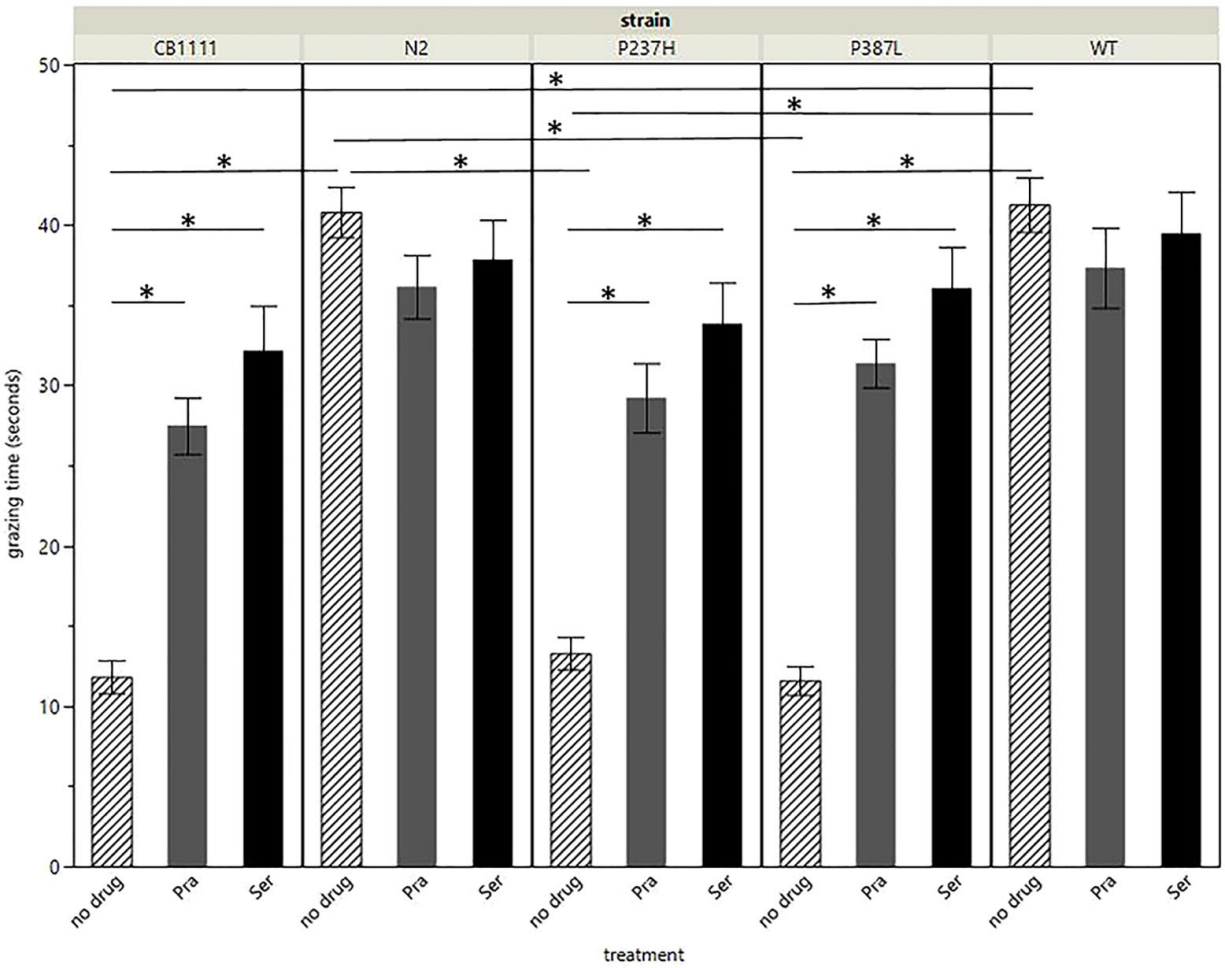
**Grazing results for each group.** Bars represent the time taken (seconds) to move onto the bacterial lawn, either with no drug treatment, 5 mM pramipexole (Pra) or 5 mM serotonin (Ser). *N*≥30 for each group. Error bars: s.e.m. Asterisks represent *P*<0.0001.

Linear model analysis revealed significant effects of strain (ANOVA *P*<0.0001, F-ratio 28.7) and treatment (ANOVA *P*<0.0001, F-ratio 29.7) on grazing. There was no effect of trial (ANOVA *P*=0.802, F-ratio 0.22; trials were performed on three separate occasions) on grazing. *Post-hoc* analysis of the *SLC18A2* genotype revealed significant effects on grazing (Fig. 2 and Table S1), with the *cat-1* knockout strain (CB1111) having a significantly reduced grazing time (mean 11.8 s, s.e.m. 1.03 s) compared to the N2 strain (mean 40.8 s, s.e.m. 1.57 s, Tukey HSD *P*<0.0001). This is indicative of reduced food recognition in the absence of VMAT. Importantly, there was no significant difference in grazing time between the WT human *SLC18A2* transgenic (mean 41.3, s.e.m. 1.70 s; Tukey HSD *P*=0.98) and the N2 *C. elegans*, indicating that transgenic expression of human *SLC18A2* at the ttTi5605 locus can restore the grazing deficit seen in CB1111 *cat-1* knockout *C. elegans*. Comparisons between the WT strain and the P237H transgenic (mean 13.3 s, s.e.m. 1.02 s, Tukey HSD *P*<0.0001) and P387L transgenic (mean 11.6 s, s.e.m. 0.90 s, Tukey HSD *P*<0.0001) demonstrated that both mutations cause significant grazing impairment, reducing it to levels comparable with the *cat-1* knockout. Pairwise comparisons between untreated strains are shown in Table 1.

**Table 1.**
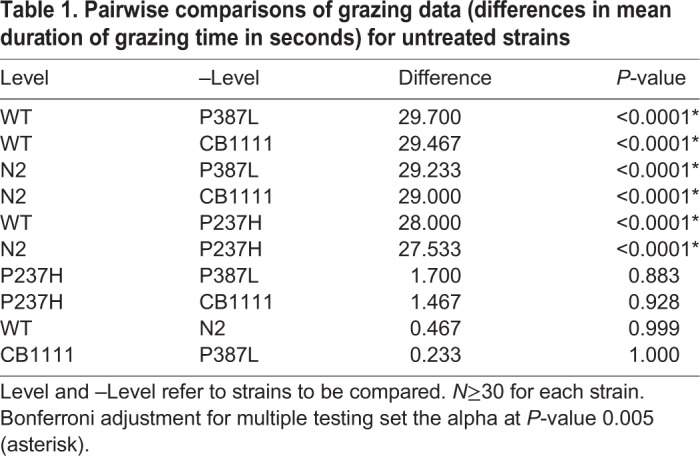
**Pairwise comparisons of grazing data (differences in mean duration of grazing time in seconds) for untreated strains**

To provide some preliminary insight into the effects of exogenous serotonin and pramipexole on *C. elegans* VMAT function, the grazing phenotype was evaluated in the presence of either 5 mM serotonin or 5 mM pramipexole. Treatment with either compound significantly improved grazing in the P237H, P387L and CB1111 strains (Tukey HSD *P*<0.0001 for each treatment across all three strains, Table 2). While serotonin consistently showed a greater effect than pramipexole (Fig. 2), this difference was not significant (Tukey HSD *P*>0.1) for any strain. There was no significant treatment effect for either drug on either of the control strains.

**Table 2.**
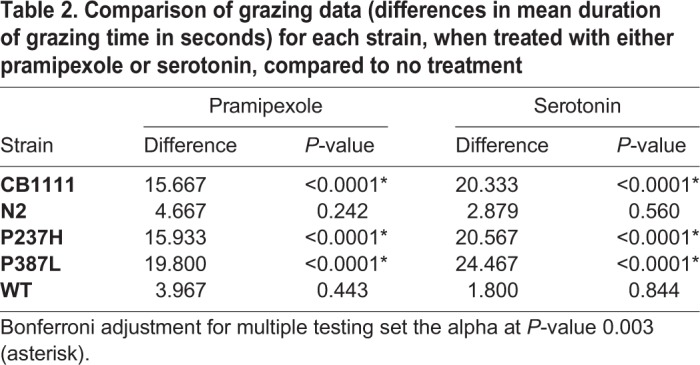
**Comparison of grazing data (differences in mean duration of grazing time in seconds) for each strain, when treated with either pramipexole or serotonin, compared to no treatment**

#### Pharyngeal pumping

**Fig. 3.**
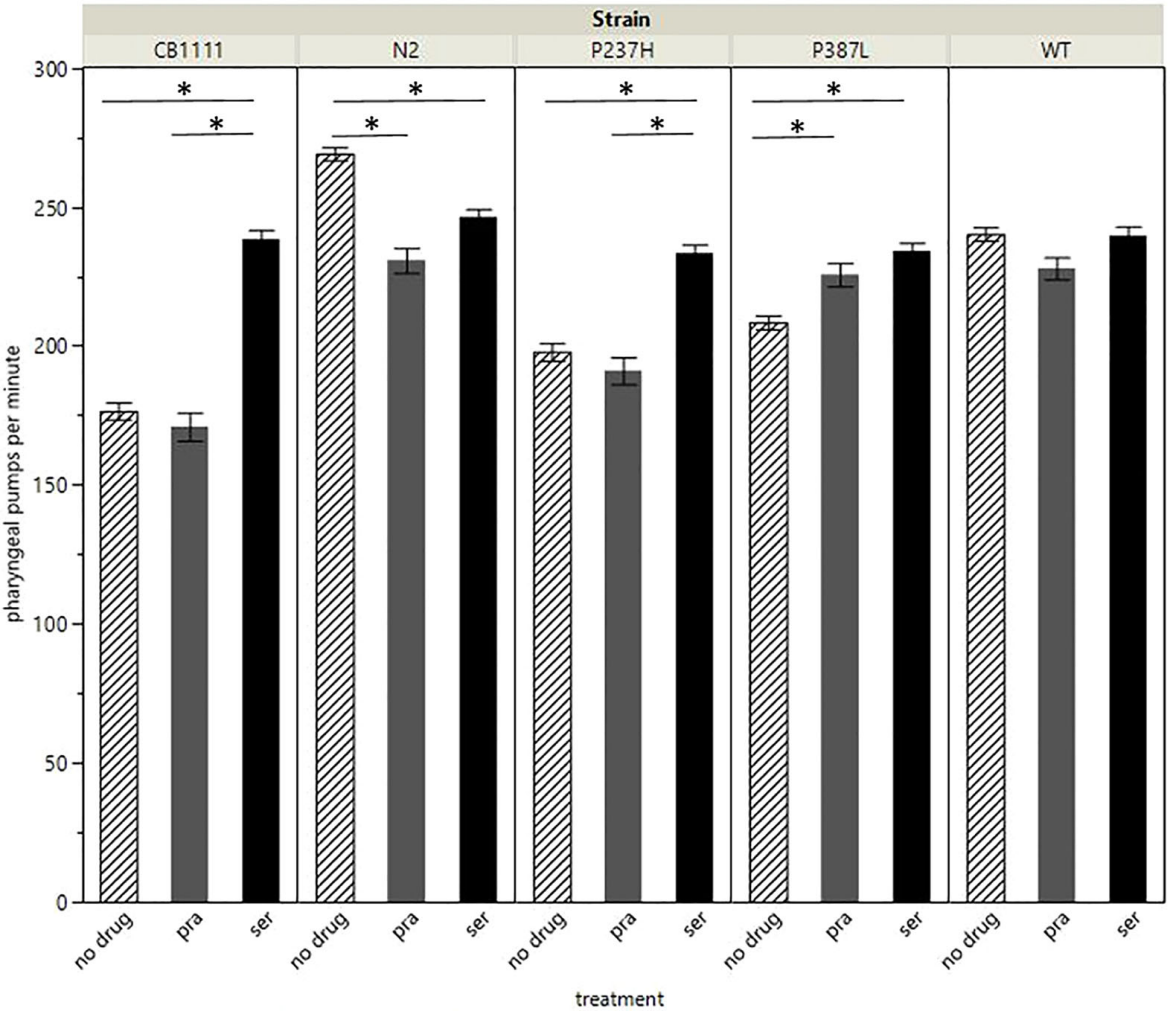
**Pharyngeal pumping results from each group.** *N*≥90 for each test without drugs. *N*=30 for each drug treatment group. Bars represent the average number of pharyngeal pumps, either with no drug treatment, 5 mM pramipexole (pra) or 5 mM serotonin (ser). Error bars: s.e.m. Asterisks represent significant differences (*P*<0.0001). For simplicity, significance for all untreated strain comparisons are not shown; all were significantly different (Tukey HSD *P*-value less than 0.003).

Pharyngeal pumping in *C. elegans* is another well-defined phenotype, dependent on serotonin signalling, and was used to investigate serotonin signalling in these models (Fig. 3 and Table S2).

Linear model analysis revealed significant effects of strain (ANOVA *P*<0.0001, F-ratio 132.2) and treatment (ANOVA *P*<0.0001, F-ratio 26.1) on pharyngeal pumping. There was no effect of trial (ANOVA *P=*0.757, F-ratio 0.27; trials were performed on three separate occasions) on grazing. *Post-hoc* analysis of the *SLC18A2* genotype revealed that all pairwise comparisons between strains were significantly altered (Tukey HSD *P*<0.005, Table 3). N2 had the greatest number of pumps per minute (mean 269, s.e.m. 2.33), and the CB1111 strain had the least (mean 177, s.e.m. 3.04). Transgenic expression of WT human *SLC18A2* (mean 240, s.e.m. 2.34) partially restored the deficit seen in CB1111 (Tukey HSD *P*<0.0001), but the value remained significantly lower (*P*<0.0001) than levels observed in the N2 strain. The mutant VMAT2 variants also displayed significantly more pumps (P237H mean 198, s.e.m. 3.2; P387L mean 209, s.e.m. 2.48), when compared to the CB1111 knockout strain (Tukey HSD *P*<0.0001 for both comparisons, Table 3). However, pumping in both mutation-carrying strains was significantly less than that observed in the WT *C. elegans*, where 69% of the difference between CB1111 and N2 levels was restored (Tukey HSD *P*<0.0001), while only a 23.1% and 34.4% rescue was achieved for the P237H and P387L strains, respectively.

**Table 3.**
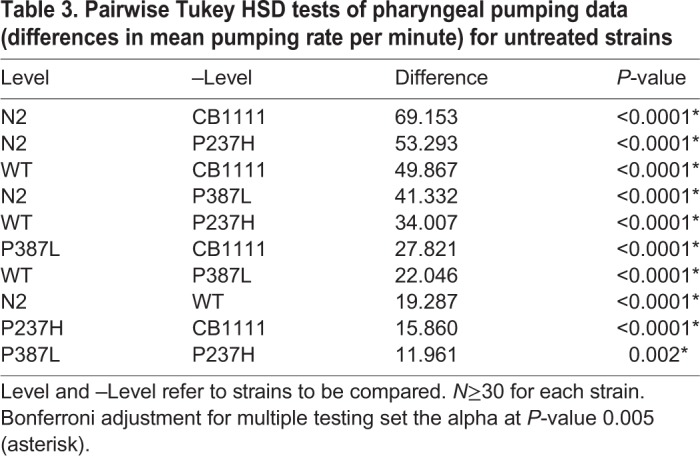
**Pairwise Tukey HSD tests of pharyngeal pumping data (differences in mean pumping rate per minute) for untreated strains**

When the pharyngeal pumping phenotype was re-assessed in the presence of either 5 mM pramipexole or serotonin (Table 4), we found that serotonin restored the mean number of pumps per minute in the CB1111 (mean 238, s.e.m. 3.4), P387L (mean 234, s.e.m. 3.01) and P237H (mean 233, s.e.m. 3.03) strains (*P*<0.0001 for all strains when compared to no drug treatment) to levels close to those seen in the WT transgenic (with or without serotonin) (Tukey HSD *P*<0.003). In addition, a more rhythmic, consistent pumping action (when compared to the untreated knockout or mutation-carrying strains) was observed with serotonin. This was not quantified, and requires further validation. Only the P387L strain significantly increased in pumping rate after addition of pramipexole (Tukey HSD *P*=0.0027), demonstrating a differentiated effect from the P237H mutation (Tukey HSD *P*=0.4712). Interestingly, pumping rate was adversely affected following treatment with both pramipexole and serotonin in the N2 strain (Table 4).

**Table 4.**
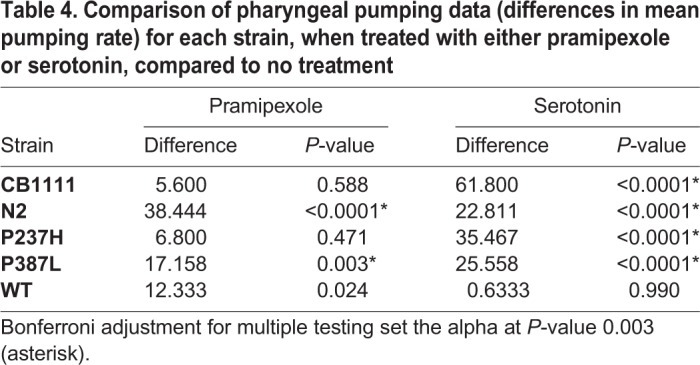
**Comparison of pharyngeal pumping data (differences in mean pumping rate) for each strain, when treated with either pramipexole or serotonin, compared to no treatment**

## DISCUSSION

We have utilised *C. elegans* as a model to validate the role of two known *SLC18A2* mutations (P237H and P387L) in brain dopamine-serotonin vesicular transport disease. While previous functional investigations of the P387L mutation revealed a severe reduction, but not complete loss, in VMAT2 transport ability (Rilstone et al., 2013), there has been no research into the consequence of the P237H mutation that has been observed in both the New Zealand and Iraqi families. We present here the first animal models of the disease. Three full-length cDNA transgenic models were developed (one for each mutation and WT human *SLC18A2* gene), and the impact of both mutations on monoamine neurotransmitter transport was assessed and compared to a previously established *C. elegans* homologue (*cat-1*) knockout model and a WT N2 strain.

It is known that VMAT2 plays an important role in dopamine and serotonin signalling; therefore, phenotypes dependent on these neurotransmitters [grazing and pharyngeal pumping (Loer and Kenyon, 1993; Sawin et al., 2000)] were evaluated in the models to provide insight into the relative function of the mutations. Results from both assays demonstrated a significant impairment in both strains carrying the human mutations, which was comparable to that of the *cat-1* knockout (CB1111). Interestingly, comparisons of the phenotypic function of the strains carrying the P237H and P387L variants suggest that the P237H mutation may have a more significant impact on protein function, which aligns with the comparative phenotypic description of the cases (Jacobsen et al., 2016; Rath et al., 2017; Rilstone et al., 2013). Further functional work assessing the impact of these mutations on protein function is required to confirm their relative effects.

The grazing assay, designed to assess food recognition by measuring the time taken for the length of the *C. elegans* to move onto the bacterial lawn (and indicative of dopamine and serotonin signalling), demonstrated a deficit in the ability to recognise food in the P237H (mean 13.3 s) and P387L (mean 11.6 s) *C. elegans* strains, despite the WT *SLC18A2* transgenic displaying normal food recognition (mean 41.3 s).

The pharyngeal pumping experiments revealed the mean pumping rate of the P237H and P387L strain to be 32% and 48% of the WT strain, respectively. It is important to consider that, although the CB1111 strain has no functional VMAT gene, the pharynx in these animals still pumps, suggesting that this is not entirely dependent on VMAT pathways. Cell culture experiments by Rilstone et al. evaluating serotonin uptake of vesicles containing mutant VMAT2 (P387L mutation) showed that the level of uptake was less than 5 pmol/mg over 10 min, compared to an uptake level of 25∼30 pmol/mg in WT VMAT2-containing vesicles (Rilstone et al., 2013). This demonstrated the level of neurotransmitter transport in the P387L mutant protein to be approximately 16-20% of normal function (compared to the 48% rescue of the pharyngeal pumping phenotype in the assay presented here). Utilising both data sets, one may postulate that a small percent of transport can provide a relatively greater level of rescue (i.e. 20% of normal transport results in a 48% rescue of phenotype). However, any conclusions from the phenotyping assays should only point to relative and not absolute effects on neurotransmitter transport. The data from the pharyngeal pumping assays presented here provide evidence that both mutations result in a very low level of protein function when compared to the WT VMAT2 protein, which is consistent with that reported by Rilstone et al.

Interestingly, grazing and phenotypic pumping were not fully restored to levels seen in N2 *C. elegans*, despite showing significant improvement relative to the CB1111 knockout strain. This is perhaps not unexpected, given that the proteins are only 49% similar (Duerr et al., 1999). It would be interesting to assess the effects of the mutations at the equivalent positions in *cat-1*.

Preliminary analysis of the therapeutic benefits of serotonin and pramipexole were assessed in these models using the established grazing and pharyngeal pumping phenotypes. Serotonin administration was successful in restoring pumping rates in the P237H, P387L and CB1111 strains, improving both the frequency (number per minute) and function (rhythmic cadence). Furthermore, serotonin administration successfully restored grazing deficits seen in the P237H, P387L and CB1111 strains. Previous work has identified successful recovery of serotonin signalling in CB1111 *C. elegans* (Loer and Kenyon, 1993), supporting this observation. The addition of pramipexole appeared to partially restore the pharyngeal pumping phenotype observed in the P387L *C. elegans* but not in the P237H or CB1111 strains, reflecting a difference in mutant protein function and suggesting that therapeutic success may be dependent on the location of the mutation. Additionally, the addition of pramipexole successfully restored grazing deficits in the P237H, P387L and CB1111 strains, providing evidence that pramipexole can rescue dopamine-dependent phenotypes in the *C. elegans* models.

Interestingly, the N2 strain displayed hyperactive movement when treated with either serotonin or pramipexole (fast backward movement, rapid twitches and increases in sporadic pumping), and were never observed to be stationary and eating. In comparison, the WT strain exhibited no adverse behaviours following drug treatment. Therefore, the improved pumping rate observed in the transgenic animals may not be caused directly by pramipexole, but indirectly via mechanisms that impact the grazing ability of *C. elegans*. Excess pramipexole may also result in over-stimulation of the *C. elegans*. Further experiments are required to determine dose effects of both serotonin and pramipexole across these strains, as well as the basal levels of these neurotransmitters and the affinity of *C. elegans* receptors for pramipexole.

A significant consideration from the case reports is that the response to treatment appears to have a lower impact in older children, as demonstrated by the greater improvement in younger patients following pramipexole treatment (Rilstone et al., 2013). Given that *C. elegans* has a life span of only a few weeks, it is hard to assess age-related effects of drug treatment and follow-up in a longer-living organism will be required. Interestingly, administration of L-DOPA to both cohorts of patients caused worsening of symptoms (Jacobsen et al., 2016; Rilstone et al., 2013). This may be due to L-DOPA-induced cytotoxicity, or apoptosis-induced effects of increased signalling on dopaminergic neurons (Jeon et al., 2010). It would be interesting to assess the effects of L-DOPA in these models.

Both the case reports and the *C. elegans* transgenics described here demonstrate that bypassing the need for vesicular transportation and directly targeting neurotransmitter receptors successfully alleviates some of the symptoms caused by the disease. Considering the potential therapeutic impacts for children harbouring mutations in the *SLC18A2* gene, the importance of an early diagnosis to optimise treatment, prevent cell damage or death and increase quality of life is clear. Based on these *C. elegans* studies, it is possible that dual administration of pramipexole and serotonin (as 5-hydroxytryptophan) may be of benefit.

### Conclusion

The *C. elegans* models described here are the first animal models developed for brain dopamine-serotonin vesicular transport disease, and provide insight into the relative biological impact of these mutations, which may ultimately help guide management and treatment options for this disease.

## MATERIALS AND METHODS

### *Caenorhabditis elegans* maintenance

Synchronised *C. elegans* populations were maintained on Nematode Growth Medium (NGM) on 35×10 mm agar Petri dishes in 15 or 20°C incubators, and were inspected daily to ensure that there was no overcrowding or contamination. *Escherichia coli* HB101 was used as a food source.

### Development of transgenic lines

**Fig. 4.**
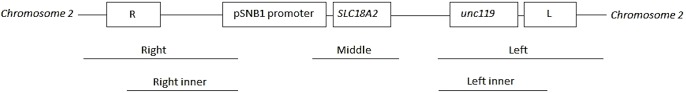
**Linear schematic showing the PCR design to verify transgenic integration in *C. elegans* chromosome 2.** PCR primer pairs used: left (unc5401+NM3880), left inner (unc5401+NM3887), middle (unc5402+seqSNB01), right inner (pSNB01+NM3888) and right (pSNB01+NM3884). R and L are homologous sites used for insertion into site ttTi5605 on chromosome 2. For a full plasmid map showing genes and primer locations, see Fig. S1.

Transgenics were generated following the MosSCI method using the *C. elegans* strain EG6699 (locus ttTi5605, chromosome 2: 0.78, genomic position: II:8420158..8420158) (Frøkjaer-Jensen et al., 2012, 2014). The plasmid consisted of a Mos1 transposon containing *SLC18A2* (full-length human cDNA) under a synaptobrevin (*snb-1*) promoter (expressed in *C. elegans* neurons and localised to synaptic regions, and used in the *cat-1* knockout transgenic generated by Duerr et al., 1999), *unc-119*(+) and homologous sites (L and R) as shown in Fig. 4. Vectors were also constructed carrying both the P237H and P387L mutations within the human cDNA (Table 5). It is unclear whether all *C. elegans* neurons express *cat-1*; therefore, there could be a potential confounding effect if the human protein is expressed in neurons in which endogenous *cat-1* is not expressed in the normal physiological state.

**Table 5.**
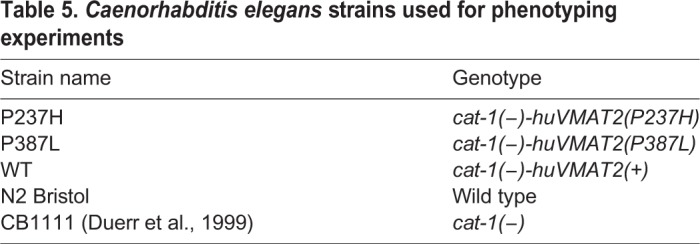
***Caenorhabditis elegans* strains used for phenotyping experiments**

Microinjection was performed using an Injectman NI 2 micromanipulator (Eppendorf) attached to a Leica inverted compound microscope. *Caenorhabditis elegans* were immobilised to slides using a 2% agarose solution. The plasmid DNA was purified using an Axygen maxiprep kit (50 ng/μl) and injected with pCFJ601 (100 ng/μl, transposase), pAM122 (10 ng/μl, peel-1 toxin), pGH8 (10 ng/μl, mCherry in neurons), pCFJ90 (2.5 ng/μl, mCherry in the pharynx) and pCFJ104 (5 ng/μl, mCherry in the body muscle). The injection cocktail was delivered at approximately 1000 psi until the mixture appeared to fill the gonad of the *C. elegans*.

Following microinjection, *C. elegans* were placed in recovery buffer (66 mM NaCl, 5 mM HEPES pH 7.2, 3 mM CaCl_2_, 3 mM MgCl_2_, 2.4 mM KCl and 4% glucose), and transferred to a seeded NGM plate and incubated at 25°C. After approximately 10 days, the plates containing the injected *C. elegans* were transferred to a 34°C incubator for 2 h, to activate expression of the toxic *peel-1* gene.

*Caenorhabditis elegans* that survived the heat-shock treatment and displayed normal movement (successful incorporation of *unc-119*) were examined under a fluorescence microscope to identify any still carrying the extrachromosomal mCherry coding plasmids. *Caenorhabditis elegans* showing no signs of mCherry fluorescence were transferred to fresh NGM plates, as they had evidently lost the extrachromosomal arrays and the desired chromosomal integration had likely occurred. The selected *C. elegans* were propagated for several generations and bred to homozygosity (demonstrated by loss of the uncoordinated phenotype due to rescue of *unc-119*), and confirmed by DNA analysis using PCR to identify the presence of the transgene using primer pairs specified in Table 6 (primer sequences available in Table S3).

**Table 6.**
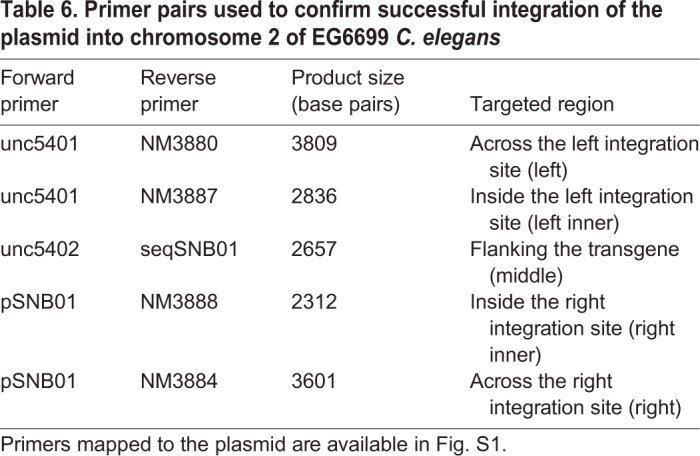
**Primer pairs used to confirm successful integration of the plasmid into chromosome 2 of EG6699 *C. elegans***

The three resulting strains containing the transgene [*huVMAT2(+)*, *huVMAT2(P387L)* and *huVMAT2(P237H)*] were crossbred with the CB1111 *cat-1* knockout strain to establish lines homozygous for the transgenes. These final strains are designated as WT, P387L and P237H (Table 5). Homozygosity was confirmed by PCR and Sanger sequencing. The males used for crossing were created by exposure to ethanol, as previously described (Lyons and Hecht, 1997). Primers used for all genotyping assays are described in Table 6, Table S3 and graphically illustrated in Fig. 4.

The N2 and CB1111 strains were provided by the Caenorhabditis Genetics Center (CGC), which is funded by NIH Office of Research Infrastructure Programs (P40 OD010440).

### Reverse transcription

RNA was extracted using a previously published single-worm method (Ly et al., 2015), and RT-PCR was used to determine successful transcription of the transgenes using the Maxima Reverse Transcription kit (ThermoFisher Scientific).

### Phenotyping assays

Synchronised adult *C. elegans* were used for all experiments, and all strains were assessed in parallel across 3 days. Synchronised populations were achieved by placing adults on plates for several hours to lay eggs. Once several eggs had been laid, the adults were removed. The *C. elegans* were housed at 20°C prior to phenotyping, and assessed in a temperature-controlled room also at 20°C. Bacterial lawn thickness and dryness of the agar was visually inspected in all assays to ensure consistency. Preliminary phenotyping involved assessing pharyngeal pumping for serotonin signalling, and grazing behaviour for dopamine and serotonin signalling, as these phenotypes have been shown to be affected by these neurotransmitters (Chase and Koelle, 2007).

Grazing activity was measured by assessing the behaviour of *C. elegans* searching for food (bacterial lawn). To perform this assay, groups of *C. elegans* were transferred to a fresh agar plate containing many small bacterial lawns (<2 μl). After encountering the bacterial lawn, the time taken for a *C. elegans* to move fully onto the bacterial lawn (1 body length) was recorded. To control for increased movement due to transfer of *C. elegans* to a new plate, any recordings of individuals encountering the lawn within 1 min of plate transfer were excluded. This is a similar method to that applied by Duerr et al. in their investigation of monoamine-dependent behaviours (Duerr et al., 1999). To measure pharyngeal pumping rate, *C. elegans* were transferred to an NGM plate with a bacterial lawn and left for 10 min to acclimate. The rate was determined by focusing on the pharyngeal muscle in individual worms under a dissecting microscope and counting each individual pump over a 60 s period, and tracking the *C. elegans* as it moved freely about the plate, as described previously (Raizen et al., 2012).

### Drug treatment

To assess treatment responses, serotonin (Sigma) or pramipexole (Sigma) were spread on top of agarose plates to a final concentration of 5 mM and given time to be absorbed. As soon as the solutions were absorbed into the agar (determined by visual inspection), the phenotyping assays were conducted.

### Statistical analysis

Statistical analysis was performed using the JMP statistical package (JMP^®^ 11.2.0), in which the individual worms within each trial were treated as biological replicates. The effect of strain, treatment and trial were assessed using an ANOVA model because data from grazing and pharyngeal pumping trials approximated a normal distribution (determined by visual inspection and Shapiro-Wilks tests). Subsequently, *post-hoc* (all pairs Tukey HSD) analysis were performed and Bonferroni adjustment for multiple testing was made.

## Supplementary Material

Supplementary information
