# Supplementary material for: Modelling brain dopamine-serotonin vesicular transport disease in Caenorhabditis elegans
Source: Dis Model Mech. 2018 Nov 9;11(11):dmm035709. doi: 10.1242/dmm.035709 (PMC6262812; doi:10.1242/dmm.035709)
Supplement: Supplementary information [file dmm-11-035709-s1.pdf]

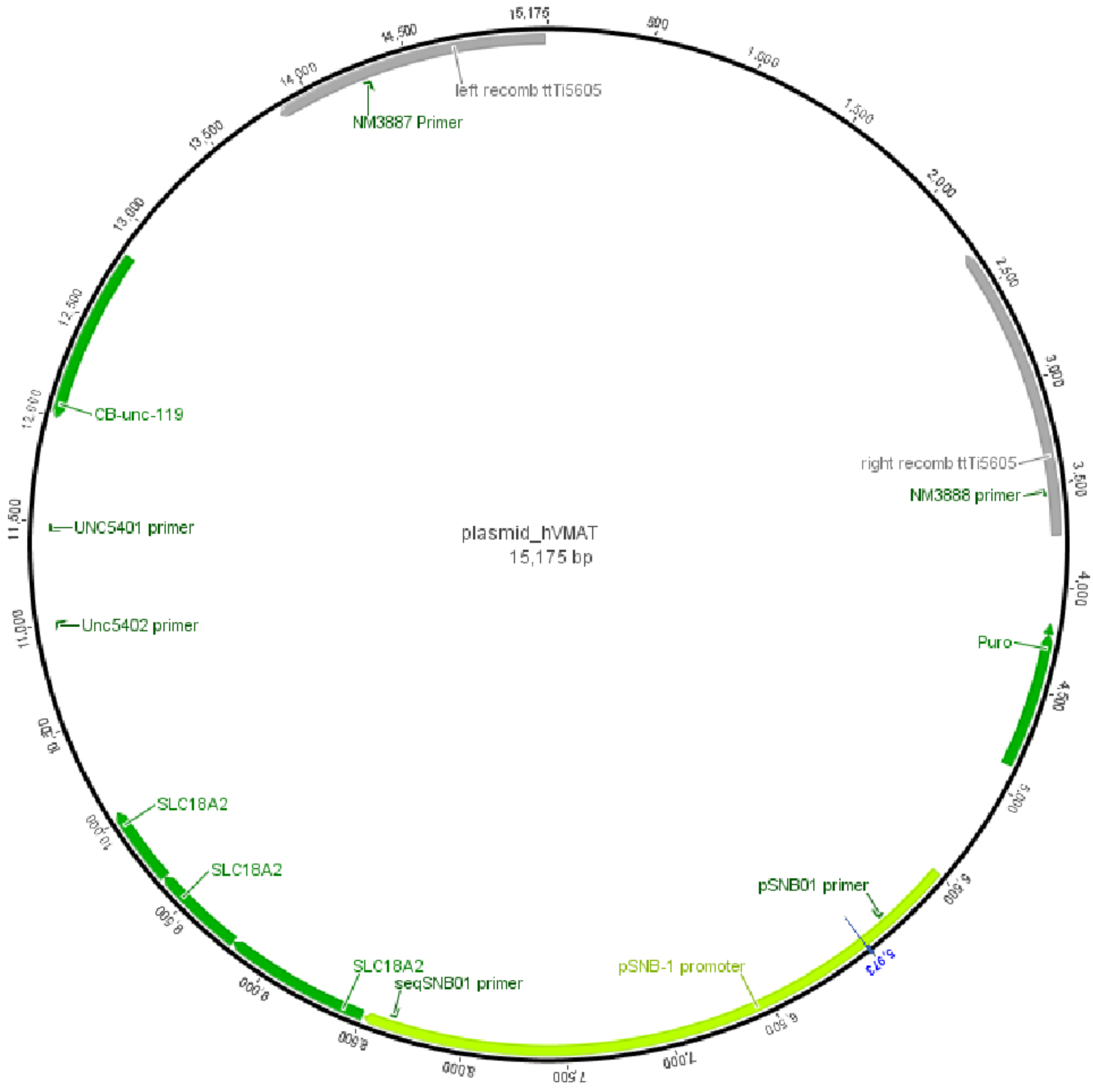

Fig. S1. The plasmid template used in microinjection. Human *SLC18A2*, a synaptobrevin promotor (pSNB-1), and *unc-119* are contained within Left and Right homologous recombination sequences. Primers are shown with arrows indicating direction.

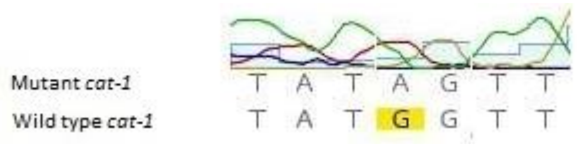

Fig. S2. Sanger traces confirming the KO *cat-1* genotype in transgenic nematodes created in this study.

Disease Models & Mechanisms • Supplementary information

Table S1. Grazing data from each strain and treatment. N = 30 for each strain in each treatment. SD: standard deviation, SE: standard error.

Strain	No drug			Pramipexole			Serotonin		
	Mean	SD	SE	Mean	SD	SE	Mean	SD	SE
CB1111	11.83	5.676	1.036	27.50	9.716	1.774	32.17	15.37	2.806
N2	40.83	8.587	1.568	36.17	10.900	1.990	37.87	13.43	2.452
P237H	13.30	5.590	1.021	29.23	11.830	2.160	33.87	14.04	2.564
P387L	11.60	4.903	0.895	31.40	8.3860	1.531	36.07	14.12	2.578
WT	41.30	9.308	1.699	37.33	13.680	2.497	39.50	14.14	2.582

Disease Models & Mechanisms • Supplementary information

Table S2. Pharyngeal pumping data from each strain and treatment. N ≥ 30 for each strain in each treatment. SD: standard deviation, SE: standard error

Strain	No drug			Pramipexole			Serotonin		
	Mean	SD	SE	Mean	SD	SE	Mean	SD	SE
CB1111	176.6	28.962	3.053	171.0	27.914	5.096	238.4	18.517	3.381
N2	269.2	22.210	2.341	230.8	24.498	4.473	246.4	16.075	2.935
P237H	198.0	30.510	3.216	191.2	26.471	4.833	233.4	16.598	3.030
P387L	208.5	27.136	2.477	225.7	22.884	4.178	234.1	16.490	3.011
WT	240.3	23.177	2.443	228.0	21.640	3.950	239.7	18.434	3.366

Table S3. Primer sequences.

Primer	Sequence (5'-3')
**unc5401**	TGTCAAGAGTTTCGAGGACG
**unc5402**	CTTTCTCCCTGTGCTCCCAC
**NM3880**	AGGCAGAATGTGAACAAGACTCG
**NM3884**	ATCGGGAGGCGAACCTAACTG
**NM3887**	ACCGGAAACCAAAGGACGAGAG
**NM3888**	ACGCCCAGGAGAACACGTTAG
**seqSNB01**	ATGCATAAATGGGCGTCGC
**pSNB01**	CGTCGGCGAGTATGTGTGTG
**SSU18A**	AAAGATTAAGCCATGCATG
**SSU26R**	CATTCTTGGCAAATGCTTTCG
**seqhVMATf1**	GCAAGTTGGTCTGTTGTTTGCC
**seqhVMATr1**	GGCATCATTGACGAATCCACC
